# Attending to global versus local stimulus features modulates neural processing of low versus high spatial frequencies: an analysis with event-related brain potentials

**DOI:** 10.3389/fpsyg.2014.00277

**Published:** 2014-04-09

**Authors:** Anastasia V. Flevaris, Antigona Martínez, Steven A. Hillyard

**Affiliations:** ^1^Department of Neurosciences, University of CaliforniaSan Diego, CA, USA; ^2^Schizophrenia Research Division, Nathan Kline Institute for Psychiatric ResearchNew York, NY, USA

**Keywords:** spatial frequency processing, global versus local attention, hemispheric asymmetry, ERPs, navon task

## Abstract

Spatial frequency (SF) selection has long been recognized to play a role in global and local processing, though the nature of the relationship between SF processing and global/local perception is debated. Previous studies have shown that attention to relatively lower SFs facilitates global perception, and that attention to relatively higher SFs facilitates local perception. Here we recorded event-related brain potentials (ERPs) to investigate whether processing of low versus high SFs is modulated automatically during global and local perception, and to examine the time course of any such effects. Participants compared bilaterally presented hierarchical letter stimuli and attended to either the global or local levels. Irrelevant SF grating probes flashed at the center of the display 200 ms after the onset of the hierarchical letter stimuli could either be low or high in SF. It was found that ERPs elicited by the SF grating probes differed as a function of attended level (global versus local). ERPs elicited by low SF grating probes were more positive in the interval 196–236 ms during global than local attention, and this difference was greater over the right occipital scalp. In contrast, ERPs elicited by the high SF gratings were more positive in the interval 250–290 ms during local than global attention, and this difference was bilaterally distributed over the occipital scalp. These results indicate that directing attention to global versus local levels of a hierarchical display facilitates automatic perceptual processing of low versus high SFs, respectively, and this facilitation is not limited to the locations occupied by the hierarchical display. The relatively long latency of these attention-related ERP modulations suggests that initial (early) SF processing is not affected by attention to hierarchical level, lending support to theories positing a higher level mechanism to underlie the relationship between SF processing and global versus local perception.

## INTRODUCTION

**FIGURE 1:**
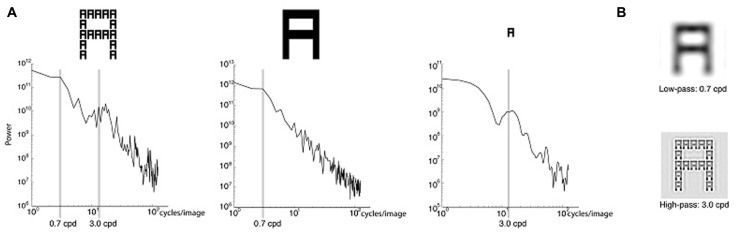
**(A)** Plots showing the spatial frequency (SF) spectrum of the Navon displays and the global versus local letters. Power is shown for SFs in cycles/image. The SFs used in the experiment – 0.7 cycles/degree (cpd) and 3 cpd – are highlighted. The left plot shows the SF spectrum for Navon displays used in the experiment. The middle plot shows the SF spectrum for a single letter the same size as the global letter in the Navon stimulus, constructed by filling in the local letters. The right plot shows the SF spectrum for a single local letter. **(B)** Navon displays spatially filtered to remove SFs above 0.7 cpd (top) and below 3 cpd (bottom).

**FIGURE 2:**
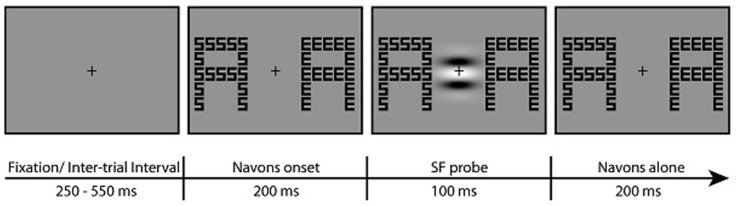
**Example stimuli for one trial.** Participants attended to the global or local level of two Navon displays at the left and right of fixation and pressed a button each time the letters at the attended level were the same in the two displays (8% of trials). In the example, a button press would be the correct response for the local but not global attention task. In 2/3 of the trials, an irrelevant SF grating probe was flashed in the center of the screen 200 ms following the onset of the Navon stimuli. The probe was either LSF or HSF (LSF probe shown in the example). Participants were instructed to ignore the probe.

Our visual environment is hierarchically organized. For example, scenes are composed of objects such as trees and houses, which are composed of more local objects such as leaves and windows, and so on. Representing elements at multiple levels of structure and flexibly directing attention to both global and local levels is vital for accurately perceiving and interacting with the visual world. Previous investigations of the neural mechanisms underlying global versus local processing have established functional hemispheric differences in extrastriate, occipito-temporal, and posterior parietal regions, with right hemisphere regions demonstrating an advantage for processing global aspects of a stimulus, and left hemisphere regions demonstrating an advantage for processing local aspects. Studies of global versus local processing have typically employed hierarchical “Navon” stimuli (Navon, 1977), in which a series of local objects (often letters, though studies have shown similar results using non-letter shapes, e.g., Kimchi and Merhav, 1991) are spatially arranged to form a global object (see **Figures 1** and **2** for examples). Hemispheric differences in global/local processing were originally demonstrated in behavioral hemifield studies, showing that participants were facilitated (i.e., faster and more accurate) at identifying global targets in a Navon stimulus that was projected first to the right hemisphere (i.e., presented in the left visual field), and they were facilitated at identifying local targets in a Navon stimulus that was projected first to the left hemisphere (Martin, 1979). The right hemisphere dominance for global processing and left hemisphere dominance for local processing has since been corroborated by neuropsychological (Delis et al., 1986; Robertson and Delis, 1986; Robertson et al., 1988, 1993; Lamb et al., 1989; Robertson and Lamb, 1991), electrophysiological (Heinze and Munte, 1993; Han and Chen, 1996; Heinze et al., 1998; Proverbio et al., 1998; Han et al., 2000, 2002; Yamaguchi et al., 2000; Malinowski et al., 2002; Volberg and Hübner, 2004; Jiang and Han, 2005) and functional imaging (Fink et al., 1997; Martinez et al., 1997; Han et al., 2002; Weissman and Woldorff, 2005) studies. The specific brain regions implicated in global/local processing have varied across studies, however, and not all studies have found the respective hemispheric differences (e.g., Polster and Rapcsak, 1994; Johannes et al., 1996; Fink et al., 1997; Heinze et al., 1998).

A parallel hemispheric asymmetry has been demonstrated for attending to, identifying, and discriminating spatial frequencies (SFs), with the right hemisphere demonstrating a preferential bias toward lower SFs (LSFs), and the left hemisphere demonstrating a higher SF (HSF) bias (e.g., Kitterle et al., 1990, 1992; Christman et al., 1991; Kenemans et al., 2000; Martinez et al., 2001). As first suggested by Broadbent (1977), a number of studies have provided evidence that differences in processing global versus local elements might be related to the underlying properties of visual-sensory pathways (“channels”) involved in processing LSFs versus HSFs, respectively (e.g., Shulman et al., 1986; Shulman and Wilson, 1987; Hughes et al., 1990; Robertson, 1996; Han et al., 2003; Boeschoten et al., 2005; Flevaris et al., 2010, 2011b, 2011a,b). For example, Shulman et al. (1986) and Shulman and Wilson (1987) demonstrated that directing attention to global objects facilitated LSF detection and directing attention to local objects facilitated HSF detection. Studies of event-related brain potentials (ERPs) elicited by Navon stimuli have shown a reduction in the ERPs elicited during global attention when the stimuli were spatially filtered or contrast balanced to remove LSFs, but not when the stimuli were spatially filtered to remove HSFs, further suggesting that global processing relies on LSFs (Han et al., 2003; Boeschoten et al., 2005). There is abundant evidence that different SFs are analyzed in parallel channels of the visual system during early stages of visual information processing (Campbell and Robson, 1968; Campbell and Maffei, 1970; Maffei and Fiorentini, 1977; Graham, 1981; De Valois and De Valois, 1988; Hughes et al., 1996); some theories therefore proposed that the two hemispheres might receive different SF inputs (or asymmetrically emphasize different inputs) from lower-level visual areas and thus differ in absolute SF biases (e.g., Sergent, 1982; Jacobs and Kosslyn, 1994). However, hemispheric asymmetries in LSFs versus HSFs were only found in tasks that required the output of multiple SF channels to be compared, (e.g., in a discrimination task but not in a detection task), suggesting that the hemispheres did not differ in an early mechanism of low-level SF processing (e.g., Kitterle et al., 1990, 1992; Christman et al., 1991).

Ivry and Robertson (1998) and Robertson and Ivry (2000) suggested that attentional selection of *relative* SF scale (i.e., the relative LSF/HSF ratio) could underlie global versus local processing. This theory proposed that a high-level attentional process interacts with low-level SF processing to support global versus local perception. In support of this theory, Flevaris et al. (2011a) demonstrated that global versus local attention affected subsequent performance in identifying LSFs versus HSFs, respectively, in a compound grating that contained both LSF and HSF components. After attending to the global structure of a Navon display, participants were faster at detecting orientation changes of the LSF component of the grating. Similarly, after attending to the local structure, participants were faster at discriminating the orientation of the HSFs. Critically, this bias was determined by the relationship between the two SFs in the compound grating (i.e., their relative SF) rather than by the absolute SF values; global attention facilitated selection of a 1.8 cycle/degree (cpd) SF when it was the relatively LSF component in the compound (i.e., paired with a higher SF), whereas local attention facilitated selection of the same 1.8 cpd SF when it was the relatively HSF component in the compound (i.e., paired with a lower SF). A subsequent EEG study showed that preparatory neural activity to an upcoming Navon display was modulated by the previously attended SFs in a compound grating, providing further evidence that a high-level, top-down influence on low-level SF processing underlies global versus local perception (Flevaris et al., 2011b).

In the present study we used scalp-recorded ERPs to investigate automatic neural processing of SF during global and local attention and to examine the time course (i.e., “early” versus “late”) of the effects on SF processing produced by global versus local attention. Previous studies have suggested that hemispheric asymmetries in global versus local perception arise during later stages of visual processing. For example, a meta-analysis found that hemispheric differences in visual hemifield studies were more pronounced when the letters at the global and local letters were incongruent (Van Kleeck, 1989), suggesting that hemispheric differences emerge after initial sensory processing of the stimulus. These results were later supported by EEG studies also showing that response conflicts were important for eliciting hemispheric asymmetries (e.g., Malinowski et al., 2002; Volberg and Hübner, 2004). Additionally, studies of ERPs elicited by Navon stimuli during global versus local attention have typically found hemispheric differences to emerge between 200 and 400 ms following stimulus onset, whereas the early sensory evoked potentials did not differ between the two hemispheres (e.g., Heinze and Munte, 1993; Heinze et al., 1998; Proverbio et al., 1998; Yamaguchi et al., 2000; Volberg and Hübner, 2004; Jiang and Han, 2005). Similarly, ERP studies of attention to SF did not find attentional modulations on the earliest sensory evoked potentials for either LSF or HSF gratings, but attentional modulations were observed later, after 150 ms (Martinez et al., 2001; Baas et al., 2002). Finally, previous ERP studies demonstrated that removing LSFs from Navon stimuli resulted in reductions in later ERPs (~190–250 ms) during global processing, but did not influence the early sensory evoked potentials (Han et al., 2002, 2003; Boeschoten et al., 2005).

While the studies reviewed above provide compelling evidence that the observations linking global versus local perception with LSF versus HSF processing, respectively, reflect a high-level mechanism at a relatively late stage of perceptual processing, studies to date have not explicitly examined automatic neural processing of SFs during global and local attention. To this end, we compared ERPs elicited by task-irrelevant LSF and HSF gratings that appeared while participants attended to the global versus local levels of Navon stimuli. These SF gratings (“probes”) appeared 200 ms after the onset of the Navon stimuli to give participants enough time to view the Navon stimuli and begin processing the relevant hierarchical level. The probes were thus assumed to occur *concurrently* with global/local attention. That is, we presented the SF grating probes only 200 ms after the onset of the Navon displays to “catch” participants during an active state of global versus local processing; differences in the ERPs elicited by the SF probes during this state of active processing were thus assumed to directly reflect differences in neural processing resulting as a consequence of global versus local processing. The assumption was that if a higher-level mechanism controlling SF selection underlies global versus local processing, then this would be reflected in the automatic perceptual processing of the irrelevant SF probes as participants focused attention on the global versus local levels. Specifically, if global processing requires attentional selection of the relatively lower SFs in an attended display, (Ivry and Robertson, 1998; Robertson and Ivry, 2000; Flevaris et al., 2011a,b), then attending to the global level of a spatially unfiltered display should engage LSF visual “channels” and automatically enhance processing of the LSF probe. Conversely, if local processing requires attentional selection of relatively higher SFs, then attending to the local level should engage visual “channels” that process HSFs and enhance processing of the HSF probe. A critical test of this hypothesis is thus to examine SF processing while participants attend to global versus local levels of spatially *unfiltered* displays, unlike prior ERP studies of the relationship between global and local processing and the processing of SF that spatially filtered the displays (e.g., Hughes et al., 1990; Han et al., 2003; Boeschoten et al., 2005). Additionally, we presented the SF probe in a physically distinct spatial location (i.e., fovea) than the Navon stimuli (i.e., periphery) to perform a stringent test of the hypothesis LSF/HSF visual “channels” are automatically enhanced during global/local attention, respectively.

Previous studies have shown that ERPs elicited by LSF and HSF gratings are markedly different, particularly in the early sensory-evoked components between 60 and 140 ms (e.g., Kenemans et al., 2000; Martinez et al., 2001; Baas et al., 2002). HSFs (3 cpd and higher) typically elicit an early C1 component (~55–100 ms), thought to be generated in the primary visual cortex (Di Russo et al., 2002), whereas LSFs (<1 cpd) do not elicit a C1, but instead elicit an early P1 component (~80–150 ms) thought to be generated in extrastriate cortex (Mangun et al., 1993; Clark et al., 1994; Heinze et al., 1994; Clark and Hillyard, 1996; Bruin et al., 1998). Studies investigating attention to SF have found earlier ERP latencies for LSFs than HSFs in both the P1 component and in the later occipital “selection negativity” (~150–250 ms) elicited in ventral extrastriate visual cortex by attended relative to unattended SFs (e.g., Skrandies, 1984; Baas et al., 2002). The fact that these extrastriate ERP components elicited by LSFs onset earlier than those elicited by HSFs is consistent with the well-known differences in integration time and response speed between LSF and HSF channels (e.g., Breitmeyer, 1975; Breitmeyer and Ganz, 1977; Schiller et al., 1979; Livingstone and Hubel, 1988; Tobimatsu and Celesia, 2006). Specifically, LSFs are processed by the faster magnocellular visual pathway, whereas HSFs are processed by the slower parvocellular visual pathway.

Given the differences in ERPs elicited by LSFs and HSFs, we compared ERPs elicited during global versus local attention separately for each SF. To determine the stage(s) of visual processing in which SFs are used to support the perception of global versus local objects, we examined both early sensory components (i.e., the C1 and the P1) as well as later ERPs that could be influenced by feedback. Based on previous ERP studies, we expected hemispheric differences to arise between 200 and 400 ms (Heinze and Munte, 1993; Heinze et al., 1998; Proverbio et al., 1998; Yamaguchi et al., 2000; Martinez et al., 2001; Baas et al., 2002; Volberg and Hübner, 2004; Jiang and Han, 2005). However, unlike previous studies examining ERPs elicited by attended stimuli, since the SF gratings were task-irrelevant and unattended in the present study, we did not expect to find an occipital selection negativity.

## MATERIALS AND METHODS

### PARTICIPANTS

Fourteen undergraduates (nine female) between the ages 18–22 from the University of California, San Diego participated in the experiment for monetary compensation. All were right handed and had normal or corrected-to-normal vision. All gave informed consent as approved by the committee for the protection of human subjects at the University of California, San Diego, and in accordance with the Declaration of Helsinki.

### STIMULI

During testing participants maintained fixation on a central white fixation cross subtending 0.5° that remained on the video screen throughout the experiment. The Navon displays consisted of black letters presented on a gray background. Seen from a distance of 60 cm, each local letter subtended 0.9° of visual angle. The local letters were positioned in a 5 × 5 grid to form a global letter that was 4.3° wide and 5.4° high. The letters were A, E, H, and S, in all their global and local combinations with the exception of congruent combinations, in which the same letter was present at the global and local level (e.g., a global A made up of local As). We excluded congruent stimuli because prior studies suggested that hemispheric differences predominately arise for incongruent stimuli (e.g., Volberg and Hübner, 2004; Hübner and Volberg, 2005). The SF grating probes were horizontally oriented Gabors subtending 6° and Gaussian-windowed with 1° standard deviation. They were cropped in width to measure 3° wide and 6° high, to prevent overlap with the peripherally presented Navon stimuli. The lower SF (LSF) probe was 0.7 cpd and the higher SF (HSF) probe was 3 cpd. These SFs corresponded to two peaks in the SF spectrum of the Navon displays (**Figure 1A**, left). Additionally, the SF spectrum of a large letter (i.e., constructed by filling in the local letters of the Navon stimulus to leave only the global letter) included a spectral peak at 0.7 cpd (**Figure 1A**, center), while the SF spectrum for a single local letter of the Navon display showed a distinct peak at 3 cpd (**Figure 1A**, right). The LSF (0.7 cpd) and HSF (3 cpd) used here were thus hypothesized to be relatively more important for resolving the global and local levels, respectively (**Figure 1B**).

### PROCEDURE

Trial timing was controlled by Presentation (Neurobehavioral Systems, Albany, CA, USA) and is depicted in **Figure 2**. Participants were instructed to maintain fixation on the central cross throughout each block of trials. On each trial, two Navon stimuli appeared bilaterally, 3° to the left and 3° to the right of the fixation cross. At the beginning of each block, participants were instructed to attend either to the global or to the local letters throughout the upcoming block and indicate via button press each time the letters at the attended level were the same in the left and right Navon displays. The order of attention task (global/local) was counterbalanced across participants. Letters at the attended level were the same in 8% of the trials. Letters at the global and local levels of each Navon display were consistent (i.e., were both the same or both different) in half of the trials; in the other half of the trials the letters at each level were inconsistent (i.e., letters at one level were the same while the letters at the other level were different).

The SF probe was flashed in the center of the screen 200 ms after the onset of the Navon displays. Participants were instructed to ignore the SF probe, which was presented for a duration of 100 ms. The probe was LSF in one third of the trials, HSF in one third of the trials, and in the last third of the trials no probe was presented during the presentation of the Navon displays. Such “no-probe” trials were used to isolate the neural activity elicited by the LSF and HSF probes. That is, ERPs elicited by the Navon displays on the “no- probe” trials were subtracted from the ERPs elicited by the Navon displays plus the SF probes to get a discrete measure of probe-related activity separate from the activity elicited by the Navon displays (see ERP analysis for details). Though the SF probes were presented in close temporal proximity to the Navon displays, effects of habituation or repetition suppression (i.e., from the Navon displays to the SF probes) were minimized by the distinct nature of the two stimulus types and by presenting the SF probe in a distinct spatial location from the Navon stimuli. The Navon displays remained on the screen for 200 ms after the offset of the SF probe, for a total of 500 ms. There was an inter-trial interval between 250 and 550 ms, yielding a presentation rate of Navon displays between 750 and 1050 ms.

Participants were given 50 practice trials at the start of each attention task, followed by 1728 test trials in each attention condition. This resulted in 576 presentations of each type of probe (i.e., LSF, HSF, no-probe) for each attention condition. Participants were given short breaks every 3 min to minimize fatigue.

### EEG RECORDING

The electroencephalography (EEG) was recorded continuously using 64 Ag-AgCl electrodes mounted on an elastic cap (Electro- Cap International, Eaton, OH, USA) according to the extended 10–20 system, and from two additional electrodes placed at the right and left mastoids. The electrode impedances were kept below 5 kΩ. Scalp signals were amplified by a battery-powered amplifier (SA Instrumentation, Encinitas, CA, USA) with a voltage gain of 10,000 (i.e., the ratio of voltage in versus out) and band-pass filtered from 0.1 to 80 Hz. Eye movements and blinks were monitored by horizontal (attached to the external canthi) and vertical (attached to the infraorbital ridge of the right eye) EOG recordings. A right mastoid electrode served as the reference for all scalp channels and the VEOG. Left and right HEOG channels were recorded as a bipolar pair. Signals were digitized to disk at 250 Hz. Each recording session lasted 90–150 min, including setup time and cap/ electrode preparation. Short breaks were given every 3 min to alleviate participant fatigue.

### ERP ANALYSIS

Trials were discarded if they contained an eye blink or an eye movement artifact (>200 μV), or if any channel exceeded 55 μV. On average, 18% of trials were rejected due to these artifacts. Averaged mastoid-referenced ERPs were calculated off-line as the difference between each scalp channel and an average of the left and right mastoid channels. To analyze neural activity to the probes, the ERPs time-locked to LSF, HSF, and “no-probe” events were averaged separately, baseline corrected from -100 to 0 ms, and low-pass filtered at 30 Hz. ERPs elicited the no-probe were subtracted from ERPs to each probe (LSF, HSF) separately for each attention condition (global, local). This subtraction procedure relies on an underlying assumption of additivity (e.g., McCarthy and Donchin, 1981) and has been used by previous ERP studies and neuroimaging studies to isolate neural activity of a given stimulus occurring within close proximity to a prior stimulus (e.g., Petersen et al., 1992; Luck et al., 1993; Flevaris et al., 2013). The assumption is that the ERPs elicited by the Navon stimuli will not change depending on the presence of the probe, so probe-related activity can be isolated by subtracting out the ERP activity elicited by the Navon displays. Following the subtraction procedure, ERPs elicited by the LSF and HSF probes were first compared via Analyses of Variance (ANOVAs) with SF (LSF, HSF), response consistency (same response at global and local levels, different response), attention (global, local) and hemisphere (right, left) as factors. Separate analyses were then performed on the LSF and HSF probes in order to compare physically identical stimulus conditions (Pitts et al., 2011; Flevaris et al., 2013), and because LSFs and HSFs elicit markedly different ERP waveforms (e.g., Kenemans et al., 2000; Martinez et al., 2001; Baas et al., 2002). To circumvent the multiple testing problem (Oken and Chiappa, 1986), we used a region of interest (ROI) based approach, in which ERPs were averaged across a number of electrodes to yield one value for the left hemisphere and one value for the right hemisphere. These regions of interest encompassed occipital electrodes (PO3/4, PO7/8, O1/2, I3/4, I5/6, SI3/4), and separate analyses were carried out on several components to examine both early (~50–180 ms) and later (~200–300 ms) SF processing as a function of attention condition. Specifically, neural activity was averaged across all left hemisphere and right hemisphere electrodes to yield one value for each hemisphere at each time window. The time windows for component measurements were centered on the maximum amplitude of the component of interest in the grand average. The length of each window was 40 ms to include a substantial portion of each component, and to account for variability in peak latencies across individual ERPs. Components were measured as mean amplitudes over the indicated time window. Follow-up planned comparisons were conducted when appropriate.

## RESULTS

### BEHAVIORAL PERFORMANCE

Responses to target stimuli (i.e., in which the letters at the attended level were the same in the right and left Navon displays) made between 200 and 1000 ms after stimulus onset were considered “hits.” Responses made to non-target stimuli (i.e., displays that were different at the attended level) in this same time interval were considered “false alarms.” This time interval was chosen because a new Navon display was presented every 750–1050 ms, so any responses made after this interval could have been made in response to the subsequent display.^1^ The average hit rate was 61 and 62% for global and local targets, respectively. The mean false alarm rate was 1 and 0.9% for global and local non-targets, respectively. Neither hit rates nor false alarms differed between the global and local attention tasks. We also calculated d-prime (Macmillan and Creelman, 1990) and did not find a difference between sensitivity in the global (2.6) versus local (2.8) tasks [*t*(13) = 1.3, ns]. Mean reaction time (RT), on the other hand, did differ between the two attention tasks [*t*(13) = 2.4, *p* = 0.04], with slightly faster RTs in response to global (652 ms) than local (678 ms) targets.

### ERP RESULTS

**FIGURE 3:**
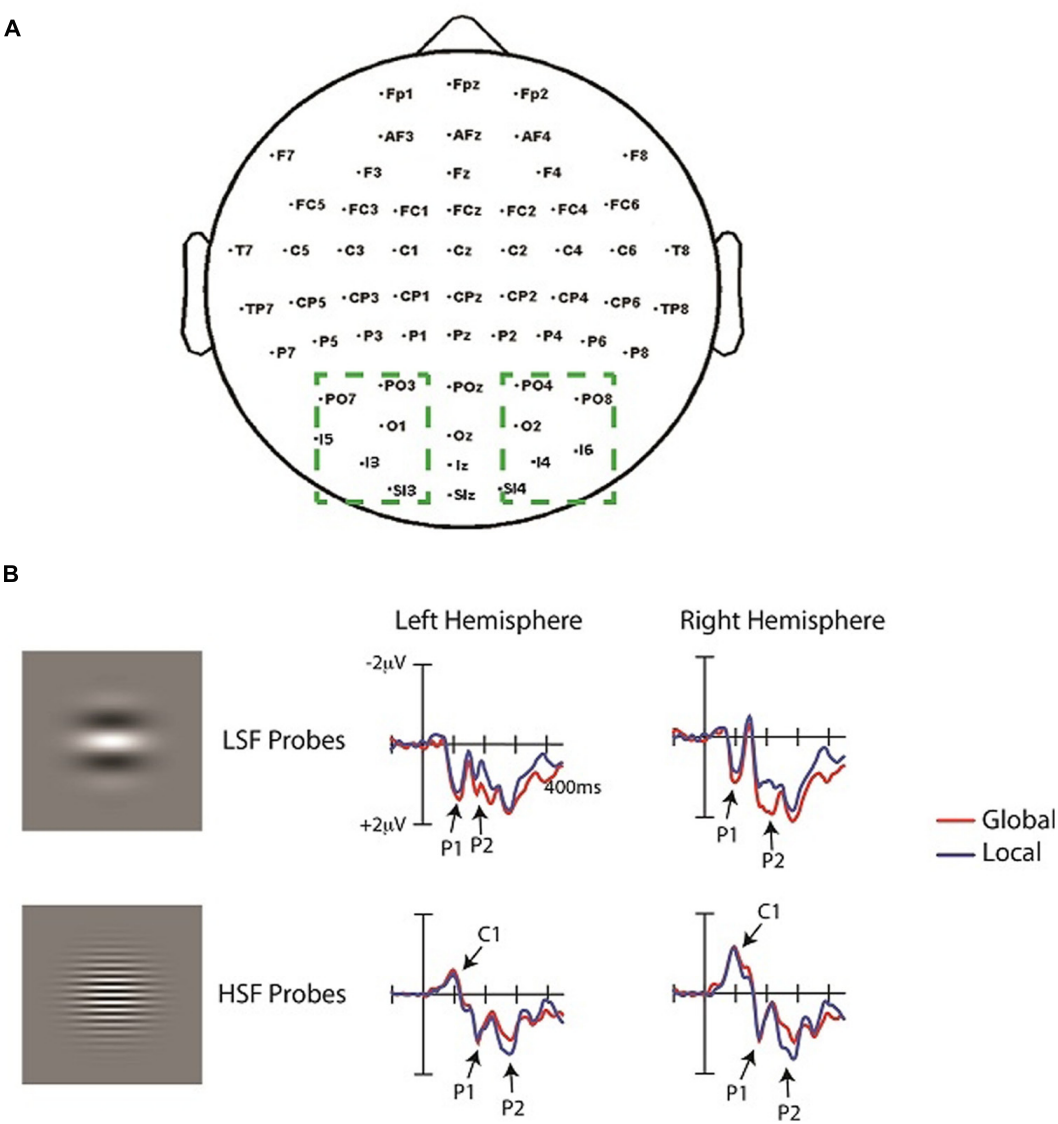
**(A)** Topography of electrodes used in the experiment. Dashed boxes show the occipital electrodes averaged in the analyses. Left hemisphere (LH) and right hemisphere (RH) electrodes were averaged separately to yield two values (one for the LH and one for the RH) in each analysis. **(B)** ERPs time-locked to the onset of the HSF and LSF probes averaged over the left and right occipital ROIs (see text) under conditions of attend-local and attend-global. ERPs elicited by the corresponding “no-probe” have been subtracted from the EPRs elicited by each probe.

The ERPs elicited by LSF and HSF probes are depicted in **Figure 3**. Consistent with prior reports (e.g., Kenemans et al., 2000; Martinez et al., 2001), LSF and HSF probes elicited very distinct waveforms. The earliest component evoked by the HSF probes was the well-documented negative C1 (75–115 ms), followed by a positive P1 component (140–180 ms). In contrast, LSF probes did not elicit a C1, but elicited an earlier P1 (80–120 ms) than HSF probes. Attention (global, local) by hemisphere (left, right) ANOVAs of the average ERP amplitudes in these time windows did not yield any significant effects, neither for LSF nor for HSF probes. For LSF probes, the analysis of P1 amplitudes revealed a trend toward an effect of attention, with slightly larger P1 amplitudes during global (0.9 μV) than local (0.8 μV) attention [*F*(1,13) = 4.5, *p* = 0.06]. There was no effect of hemisphere in this time window [*F*(1,13) <1], nor was there an interaction between attention and hemisphere [*F*(1,13) = 1.6, *p* = 0.3]. For HSF probes, the analysis of C1 amplitudes revealed neither an effect of attention (*F* <1), nor of response consistency (*F* = 2.14, *p* = 0.2); nor of hemisphere [*F*(1,13) = 3.1, *p* = 0.2], and no interactions between these factors. Similarly, the overall analysis of the P1 amplitudes revealed neither an effect of SF (*F* <1), nor an effect of response consistency (*F* <1), nor an effect of attention [*F*(1,13) = 1.6, *p* = 0.7], nor an effect of hemisphere (*F* <1), and no interactions between these factors. Hence, global versus local attention did not significantly modulate the early ERP components. Indeed, visual inspection revealed the main difference between attention conditions to emerge later, in the time window of the P2 component, though the specific time window for this component differed as a function of SF. That is, similar to the P1, the P2 elicited by LSF probes (196–236 ms) was earlier than that elicited by HSF probes (250–290 ms).

**FIGURE 4:**
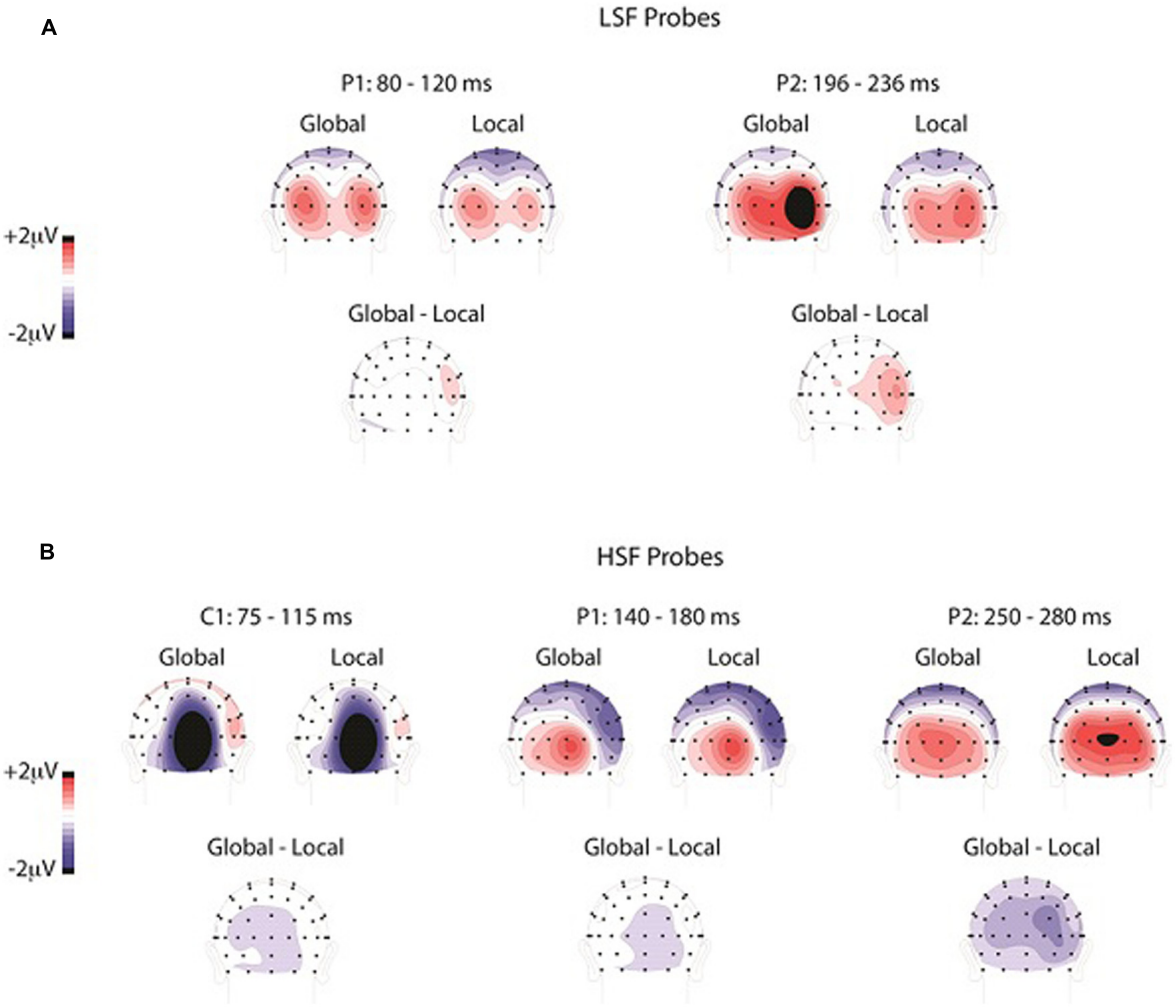
**The scalp distributions elicited by LSF (A) and HSF (B) probes during global attention, local attention, and the difference, shown during the latency ranges of the C1, P1, and P2**.

The overall ANOVA of the P2 amplitudes revealed a reliable SF by attention interaction [*F*(1,13) = 18.1, *p* < 0.001], SF by hemisphere interaction [*F*(1,13) = 10.3, *p* < 0.01], and SF by attention by hemisphere interaction [*F*(1,13) = 6.5, *p* = 0.02]. No other effects were significant. To further investigate these interactions, we analyzed the P2 elicited by LSF and HSF probes separately in order to compare the effects of global versus local attention on physically identical stimulus conditions. The attention (global, local) by hemisphere (left, right) ANOVA of the average P2 amplitudes elicited by LSF probes revealed a main effect of attention [*F*(1,13) = 9.35, *p* = 0.001], with significantly greater P2 amplitudes during global (1.6 μV) than local (1.0 μV) attention. This analysis also revealed a main effect of hemisphere [*F*(1,13) = 8.4, *p* = 0.01], with greater P2 amplitudes over the right (1.5 μV) than the left (1.1 μV) hemisphere, and an interaction between attention and hemisphere [*F*(1,13) = 5.2, *p* < 0.05]. Follow-up *t*-tests revealed that the enhancement of P2 amplitudes over the right than left hemisphere was only significant during global [*t*(13) = 3.34, *p* = 0.005] and not local [*t*(13) = 1.97, *p* > 0.05] attention. The scalp topography of the ERP in the global minus local difference wave elicited by LSF probes over the P2 time window (196–236 ms) showed a very similar distribution to that of the ERPs elicited by LSF probes during both global and local attention (**Figure 4**). To statistically evaluate whether the topographies during global and local attention differed in this time window, the amplitudes of the occipital electrodes (PO3/O4, PO7/8, O1/2, I3/4, I5/6, SI3/4) were normalized (McCarthy and Wood, 1985) to remove overall amplitude differences between attention conditions and entered into an ANOVA with attention (global, local) and electrode (1–12) as factors. This analysis did not yield an interaction between attention and electrode [*F*(11,143) = 1.7, *p* > 0.05], suggesting similar scalp topographies in the two attention conditions.

The attention (global, local) by hemisphere (left, right) ANOVA of the P2 amplitudes elicited by HSF probes also revealed a main effect of attention [*F*(1,13) = 15.3, *p* < 0.002], in this case indicating significantly greater P2 amplitudes during local (1.4 μV) than global (0.9 μV) attention. Unlike the analysis of the LSF probes, however, there was no effect of hemisphere (*F *< 1) nor an interaction between attention and hemisphere [*F*(1,13) = 1.1, *p* > 0.05] in the analysis of the HSF probes. Similar to the analysis of the LSF probes, the scalp topography of the ERP in the global minus local difference wave elicited by HSF probes over the P2 time window (250–290 ms) was very similar to that of the ERPs elicited by HSF probes during both global and local attention. Indeed, normalized ERP amplitudes (McCarthy and Wood, 1985) during this time window were entered into an attention (global, local) by electrode (1–12) ANOVA and did not yield a significant attention by electrode interaction [*F*(11,143) = 1.2, *p* > 0.05].

## DISCUSSION

In the present study participants attended to global or local levels of peripheral Navon stimuli while irrelevant SF probes flashed in the center of the display that were either relatively LSF (0.7 cpd) or HSF (3 cpd). Global versus local attention was found to influence late – and not early – sensory evoked potentials elicited by the SF probes. Although the ERPs elicited by the LSF and HSF probes differed markedly in the early evoked components in the 75–180 ms range, global versus local attention did not reliably modulate these earlier components. In contrast, ERP differences as a function of global versus local attention emerged between 200 and 300 ms for both LSF and HSF probes. LSF probes elicited an enhanced P2 (196–236 ms) during global relative to local attention, and this difference was greater over the right hemisphere. In contrast, HSF probes elicited a later P2 than LSF probes (250–290 ms), which was enhanced during local relative to global attention and bilaterally distributed over the occipital scalp.

The fact that attended level did not significantly modulate the early ERP components elicited by the SF probes suggests that low-level sensory processing of SF was not affected by attention to global versus local level. This is in line with prior studies examining the effects of selective attention to SF, which did not find attentional modulations in the earliest evoked potentials C1 and P1 (Kenemans et al., 2000; Martinez et al., 2001; Baas et al., 2002). However, the analysis of the LSF probes did reveal a trend of attention, with slightly larger P1 amplitudes during global versus local attention. Nonetheless, the attention effect on the P1 elicited by LSF probes did not reach statistical significance, and, importantly, there was no evidence of hemispheric differences in this earlier time window. The fact that the hemispheric asymmetry emerged later is consistent with the theory that the hemispheric differences in global versus local processing reflect a higher-level mechanism in SF selection rather than a low-level difference in SF biases (Ivry and Robertson, 1998; Robertson and Ivry, 2000; Flevaris et al., 2011a,b). The lack of hemispheric differences in the early evoked potentials is also consistent with previous fMRI results that found LSF and HSF processing to be symmetrically distributed across the early visual cortex (Sasaki et al., 2001).

The latency of the P2 enhancement (196–290 ms) found in the current study was within the latency range of previous global versus local attention effects (Heinze and Munte, 1993; Heinze et al., 1998; Proverbio et al., 1998; Yamaguchi et al., 2000; Volberg and Hübner, 2004; Jiang and Han, 2005), as well as previous effects of attention to SF (Kenemans et al., 2000; Martinez et al., 2001; Baas et al., 2002), although the components implicated across these studies have varied. For both LSF and HSF gratings, the scalp distributions over the P2 latency range were similar during global and local attention, suggesting that the attentional modulations on the P2 reflect the enhancement of a single perceptual process. The perceptual enhancement could reflect attentional selection of the task-relevant SF channels, supporting previous studies suggesting that attention to SFs underlies global versus local processing (e.g., Flevaris et al., 2010, 2011a,b). The SFs we used, 0.7 and 3 cpd, corresponded to SFs with spectral power in the Navon displays (**Figure 1A**); the 0.7 cpd LSF probe fell within range of relatively lower SFs in the attended Navon stimuli and the 3 cpd HSF probe fell within the range of relatively higher SFs. Hence, consistent with these prior studies, the present results support the theory that global attention involves selection of relatively lower SFs in an image, and local attention involves selection of relatively higher SFs (Ivry and Robertson, 1998; Robertson and Ivry, 2000). Specifically, the enhanced P2 elicited by the task-irrelevant LSF probes during global relative to local attention suggests that the global task required selection of relatively lower SF channels, which automatically enhanced LSF (and not HSF) probe processing. Likewise, the enhanced P2 elicited by HSF probes during local attention suggests that the local task required selection of relatively higher SF channels, automatically enhancing HSF (and not LSF) probe processing. Although global versus local attention modulated the P2 for both LSF and HSF probes, we only found hemispheric differences in the P2 elicited by LSF probes, while the P2 elicited by HSF probes was bilaterally distributed. This is in contrast to previous studies showing both a right hemisphere advantage for LSFs and a left hemisphere advantage for HSFs (e.g., Kitterle et al., 1990, 1992; Kenemans et al., 2000; Martinez et al., 2001). However, the present results are actually consistent with other findings in the global versus local processing literature that have not found hemispheric differences for both global and local tasks, but have found differences in one task or the other (e.g., Robertson et al., 1988; Weissman and Banich, 1999; Weissman and Woldorff, 2005; Flevaris et al., 2011b). As such, task by hemisphere interactions have become the gold standard for measuring hemispheric asymmetry (e.g., Hellige, 1983; Robertson et al., 1993; Weissman and Banich, 1999). Indeed, a previous EEG study found hemispheric asymmetry in the alpha band (8–12 Hz) during preparation for a global task that was modulated by attended SF in a previous compound stimulus, whereas preparation for a local task was not modulated by attended SF (Flevaris et al., 2011b). Specifically, LSF attention resulted in lower alpha band amplitudes over the right hemisphere than the left hemisphere in preparation for the global task, while there was no difference between the hemispheres following HSF attention. Alpha reduction has been assumed to reflect increased attention (e.g., Worden et al., 2000; Bastiaansen et al., 2001; Ward, 2003; Thut et al., 2006; Volberg et al., 2009; Banerjee et al., 2011; Foxe and Snyder, 2011), suggesting an enhanced attentional engagement in the right hemisphere in preparation for the global task following LSF selection. In contrast, in advance of the local task, both LSF and HSF attention resulted in greater alpha reduction over the right than left hemisphere. The global/local task in that study was similar to the one used here, in which two Navon displays were bilaterally presented in the periphery and participants determined whether the letters at the global or local levels were the same or different. It is possible that this task engages the right hemisphere to a greater degree due to the large attentional window required for both the global and local task, in line with the right hemisphere dominance in visuospatial attention (Heilman et al., 1985; Nobre et al., 1997; Bowen et al., 1999; Gitelman et al., 1999; Mesulam, 1999; Ringman et al., 2004; Becker and Karnath, 2007; Shulman et al., 2010). That is, an overall right hemisphere bias in this task could mask any underlying hemispheric differences that are greater in the left hemisphere. Future research is needed to tease out any influence of visuospatial attention on hemispheric differences to see if left hemisphere dominance emerges for HSF probes during local attention. Additionally, studies have suggested that hemispheric asymmetries are more pronounced when targets occur randomly at the global and local levels and participants must monitor both (i.e., “mixed” design), rather than requiring participants to attend to one level for an entire block of trials (i.e. “blocked” design), as we did in the present experiment (e.g., Hübner and Malinowski, 2002). We used a blocked design to investigate “top-down” directed attention to a specified level, but a mixed design may have yielded greater hemispheric differences. Importantly, in the present design there was no spatial overlap between the Navon stimuli and the SF grating probes, suggesting that low-level processing in early visual cortex could not account for the results (i.e., because low-level processing of the letter stimuli would be restricted to the spatial locations – receptive fields – they occupied). It is also unlikely that the results from this experiment can be attributed to differences in attentional window size between the global and local tasks, because participants were required to compare bilaterally presented Navon stimuli in both cases (Flevaris et al., 2011a). Hence, consistent with previous studies, the results from this experiment suggest that global versus local perception is not driven by low-level processing of SFs, but reflects the selection of relatively LSF versus HSF channels, respectively.

These data are consistent with evidence suggesting that the visual system flexibly extracts SF information in an image contingent upon task demands (e.g., Oliva and Schyns, 1997; Peyrin et al., 2005, 2006; Sowden and Schyns, 2006). For example, although LSFs are known to be processed more quickly than HSFs (e.g., Breitmeyer, 1975; Breitmeyer and Ganz, 1977; Schiller et al., 1979; Livingstone and Hubel, 1988; Tobimatsu and Celesia, 2006), evidence suggests that HSFs can be accessed earlier if they are more informative for the task (Oliva and Schyns, 1997; Schyns and Oliva, 1999). The present results support the notion that task demands flexibly modulate SF processing in the visual system because perceptual processing of SF differed as a function of task (global/local). The present results also showed that the sensory-evoked potentials elicited by LSF probes occurred earlier than those elicited by HSF probes, irrespective of task. This finding is in line with models of the visual system suggesting that, while visual analysis may start with parallel extraction of different SFs in an image, the time course of visual processing follows a predominately “coarse-to-fine” (i.e., LSF to HSF) processing strategy (e.g., Bullier, 2001; Bar, 2003; Bar et al., 2006; Hedgé, 2008; Peyrin et al., 2010). According to these models, feedback from higher level areas plays a dominant role in the computations that are carried out in earlier visual areas. Specifically, these models propose that LSFs, being processed by the faster magnocellular pathway, reach higher-order processing areas first and are then fed back into earlier visual areas to interact with and guide further processing of HSF information. In the current study, the fact that both the P1 and P2 elicited by LSF probes occurred earlier than that elicited by HSF probes is consistent with this framework. Moreover, the fact that global/local task influenced the later P2 component – and not the earlier C1 or P1 components - suggests that directed attention did not influence the initial feedforward sweep from V1 to higher-level areas, but that feedback from higher-level areas influenced SF processing. Hence, the present results demonstrate both “coarse-to-fine” feedforward processing and dynamic, top-down selection of SF scales to support global and local perception.

## Conflict of Interest Statement

The authors declare that the research was conducted in the absence of any commercial or financial relationships that could be construed as a potential conflict of interest.

## Footnotes

1 When we extended the behavioral response time interval to 200–2000 ms, the average hit rate increased to 69 and 73% for global and local targets, respectively, and the average false alarm rate also increased to 13 and 14%, respectively. The average d prime for this interval was 1.65 and 1.85 for global and local targets, respectively, but importantly, there was still no statistical difference in sensitivity between the two conditions [*t*(13) = 1.3, ns].
